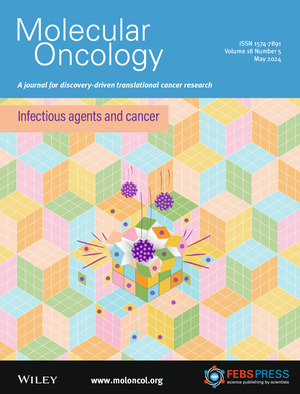# Issue Information

**DOI:** 10.1002/1878-0261.13459

**Published:** 2024-05-07

## Abstract

The issue features content focused on a variety of infectious agents directly or indirectly related to carcinogenesis. Read articles on the role of bovine meat and milk factor proteins, multispecies microbes and postbiotics in colorectal cancer in pp. 1076–1188; HPV status on cancer progression, disease severity and treatment in pp. 1209–1244; as well as the application of oncolytic viruses as cancer therapeutics in pp. 1245–1277.

On the cover: HPV integration drives oncogenesis through changes in host chromatin structure and gene‐regulation in cervical cancers. Read the full article by Singh AK, et al. in pp. 1189–1208.

Illustration credits: Mrunal Kulkarni, Anurag Kumar Singh and Nija George.